# Emotions of the Anthropocene across Oceania

**DOI:** 10.3390/ijerph19116757

**Published:** 2022-06-01

**Authors:** Rachel Clissold, Karen E. McNamara, Ross Westoby

**Affiliations:** 1 School of Earth and Environmental Sciences, The University of Queensland, Brisbane, QLD 4072, Australia; r.clissold@uq.edu.au; 2 Griffith Institute for Tourism, Griffith University, Brisbane, QLD 4111, Australia; r.westoby@griffith.edu.au

**Keywords:** climate change, eco-grief, eco-anxiety, loss and damage, mental health, Pacific Islands

## Abstract

As human activities have destabilised life on Earth, a new geological era is upon us. While there is a myriad of challenges that have emerged because of such human-driven planetary changes, one area of investigation that requires ongoing scholarly attention and scientific debate is the emotions of the Anthropocene. The emotional, mental, and psychological burdens induced by rapid and unprecedented change must be understood to better reflect the experiences of people around the globe and to initiate conversations about how emotions may be used for transformative change and effective politics. This paper aims to provide insights into the types of emotions that are emerging in Oceania as the Anthropocene unfolds. To do this, we draw on several data sets: questionnaire results with visitors of Mt Barney Lodge in the World Heritage Gondwana area in Queensland, Australia; another questionnaire with Pacific Island “experts” engaged in climate change, development, and disaster risk management work; interviews with locals living in the Cook Islands; and various spoken, written, and visual art from the Pacific. Bringing these data sets together allows us to explore a diversity of experiences, perspectives, and emotional responses to the Anthropocene from participants across Oceania. We found that acute and slow-onset weather events, experiences of direct loss and change, a perceived lack of agency or control over futures, and a sense of injustice triggered emotions including fear, stress, anxiety, exhaustion, sadness, grief, anger, frustration, helplessness, worry, but also empowerment. These results are critical for the first step of acknowledging and naming the emotions that are emerging in Oceania, such that they can then be worked through, and may be used for transformative change, effective politics, and agency over futures.

## 1. Introduction

There is little debate that our world has entered an era of significant human impacts on global systems. “Anthropocene” has been the term used to describe this period, where human activities are exerting increasing influence on the environment at all scales, and in many ways, outcompeting natural processes [1,2]. Climate change is only one big part of a much wider set of entwined catastrophes that includes the great mass extinction event, acidification of oceans, the accumulation of plastic waste, and loss of rainforests and healthy soil [3]. Perez [4] has reflected on how the Pacific Islands have been referred to as the site of “golden spikes” and anthropogenic environmental impacts, including nuclear testing, unprecedented cyclones, mass coral bleaching events, extinction of native species, the “Great Pacific Garbage Patch”, phosphate strip-mining and the destruction tied to palm oil plantations, to name a few. Scholarly engagement with the Anthropocene was originally a matter of scientific definition and debate over how to measure and project human impact in the fossil record, albeit this has evolved towards increasing engagement with the concept as a social, economic, and cultural process or phenomenon [5,6].

Head [7,8] has argued that, with the high levels of uncertainty and profound, everyday losses that the Anthropocene heralds to our environment and modern selves, we must begin to better engage with what it might mean to be a citizen of the Anthropocene—an Anthropocean. Grief, in all its complexity, diversity, and contradiction, is “a companion that will increasingly be with us” [8] (p. 13) as we experience “unimaginable scales of change” [7] (p. 313). There is and will be grief for the loss of a stable, pristine, and certain past (i.e., the Holocene), grief for the loss of conditions that underpin Western prosperity, and grief for the loss of a future characterised by hope [8]. Similarly for the Pacific context, Perez [4] has emphasised the need to examine how Pacific Islanders articulate and express feeling as they register the impacts of and life within the Anthropocene. The emotional, mental, and psychological burdens induced by rapid and unprecedented changes and the related sense of unease about our futures are already being documented across the globe [9,10,11], albeit more attention in scholarly studies and scientific debate is needed in this area [12]. These “negative” emotions and diverse manifestations of grief are widespread, profound, and cumulative [13], and need to be expressed, engaged with, and discussed more openly to better reflect the experiences of people around the globe, and to initiate conversations about how emotions may be used for transformative change and effective politics [8,12,14,15,16,17,18].

This paper aims to contribute some insights to these open discussions around emotions of the Anthropocene and public narratives of loss and mourning [8,16]. In doing so, we add to earlier discussions by providing insights into the emotions that are emerging in Oceania, and why, as the Anthropocene unfolds and forges a new reality. This is important, as research outlining how emotions are expressed, what processes they are related to, and how they are distributed remain relatively scarce in the context of climate change and other environmental problems [12]. Drawing on survey and interview data sets, as well as a body of Pacifika art, we hope to contribute to the first step of acknowledging and naming the emotions and grief in Oceania such that they can then be worked through, and the emotional energy can be reinvested in creative and productive ways.

### Literature Review: Emotional Impacts of Climatic and Environmental Change

Scholars are increasingly paying attention to the complex ways in which emotions are linked to ecological crises. While there are specific manifestations of grief that we need to acknowledge and engage with, they are part of a bigger narrative of “looming darkness” that Rose [3] (p. 215) labels “Anthropocene noir”–“the story without a known ending; the looming sense of fatality; the creeping awareness that nothing can be put right” [8]. Scholars engaging with the Anthropocene have criticised the largely unitary and undifferentiated species narrative and representation of humankind as this masks moral questions around culpability and historical responsibility [19]. In this way, it is important to remember that “We may all be in the Anthropocene but we’re not all in it in the same way” [19] (p. 8). The Anthropocene has “taken hold in plutocratic times, when economic, social and environmental injustice is marked by a deepening schism”, and where there are unequal human impacts, agency, and vulnerabilities [19] (p. 8). This is relevant for how people experience related emotional burdens and healing in the face of planetary and environmental changes in the Anthropocene, as there are many influencing and contextual factors that shape people’s emotions and processes of grieving and mourning [17,18]. There is value, therefore, in focusing on a regional context—Oceania—and providing rich, in-depth qualitative data to demonstrate the diverse emotions that emerge.

Emotions in response to ecological crises can be varied and complex, leading to several studies attempting to categorise the range of climate or eco-emotions [18,20,21,22]. Enhanced knowledge in this area is important for understanding the relationship between emotions, climate action and contextual dynamics, and to find ways of channelling emotional energy to constructive responses to climate and environmental risks [18]. Most recently, Pihkala [18] built on existing taxonomies by highlighting a broad range of emotional and mental state categories in the context of climate change: surprise-related, threat-related, sadness-related, strong anxiety-related, strong depression-related, guilt and shame-related, indignation-related, disgust-related, anger-related, envy-related, feelings of hostility and positive emotions. It is also clear that many of these emotions can be self- or other-directed [22], although these are not necessarily mutually exclusive and there can be “collective” forms of many emotions [18]. Environmentally relevant emotions also tend to overlap with “moral emotions”, such as those that are self-condemning (e.g., guilt), other-condemning (e.g., anger), other-suffering (e.g., compassion), and other-praising (e.g., elevation, awe) [22,23]. Also increasingly recognised is that, in the face of climate and ecological crises, it is not only the immediate environmental impacts that are the cause of distress, but also the resulting medium- and long-term social, economic, and cultural changes [24,25,26]. Similarly, although previous research strongly focused on the direct mental health impacts from exposure to adverse environmental conditions, the impacts of indirect exposure to climate change (e.g., media representations) are also increasingly recognised as occasioning emotional impacts [13,27].

Key and emerging areas of research include those related to grief, anxiety and worry which we will explore in more detail here [12]. There are many shades, forms, and manifestations of grief, including anger, frustration, fear, stress, hopelessness, and helplessness [17,28,29]. The most common general terms used here are “ecological grief” [30] and “climate grief” [17]. Cunsolo and Ellis [17] outline three climate-related and overlapping contexts in which grief has been reported: (1) physical ecological losses (land, ecosystems, and species), (2) disruptions to environmental knowledge and loss of identity, and (3) anticipated future ecological losses. In the context of “natural” disasters, Whittle et al. [31] have argued that emotional recovery is a critical and often “hidden” aspect of disaster recovery that can generate new vulnerabilities and can operate across timescales that are longer than expected. Albrecht’s [9,32,33] concept of “solastalgia” has also emerged in this space, which describes the homesickness one feels while still at home and, more recently, the grieving related to the loss of a healthy place and thriving ecosystem. This loss is particularly relevant for individuals and communities with strong identity ties and attachment to environment, such as for farmers or Indigenous peoples in Australia and Pacific Island communities [34,35]. This grief can also be anticipatory, emerging because of imagined futures of further loss and change, especially in relation to the environment, life, normality, health and/or prosperity but also culture, livelihoods, and ways of life [17,24,36,37].

There is also burgeoning research on and documentation of anxiety in relation to ecological and climate crises, which commonly manifest in connection with fear, grief, and guilt [28,38,39]. The increasing interest in and use of the term “eco-anxiety” refers to the stress and apprehension about anticipated threats to salient ecosystems [28], making it a form of “pre-traumatic stress disorder” [40]. Feelings of ambivalence, powerlessness and helplessness have also been common features of eco-anxiety [18,41]. Central anxieties in relation to climate change and environmental crises can relate to the sense that we are tipping into instability, the survival of our very sense of self and sense of regularity and continuity as a species, and inadequate leadership [12,42,43]. Several studies, however, also highlight the motivational aspects of eco- and climate anxiety where rather than being paralysing, anxiety helps individuals react to threats [44,45]. Others also link feelings of worry, anxiety and fear with love, care, compassion, and moral/ethical undertones, where people think about distant aspects such as people in faraway countries, animals and nature and future generations [12,18,30,46]. These are, however, “macro worries”, while some subgroups of people are likely to face direct damages and losses that produce strong emotional responses and negative mental health outcomes such as depression, anxiety, and psychological distress [12]. It is critical that people bear their anxieties to avoid driving them further underground into denial and disavowal, but also because, if we do not, “thinking deteriorates, and irrationality, lack of proportionality, hatred and narcissism are more likely to prevail” [42] (p. 46).

Most studies on the emotional impacts of climate change have focused on European, Northern American, or Australian contexts [12], and although populations that are more sensitive to weather-related phenomenon and eco-catastrophes (e.g., resource-dependent populations, people living in ecologically sensitive areas, and Indigenous Peoples) have been increasingly represented [17,25,34,47], Small Island Developing States (SIDS) experiences require more attention [48]. Focuses on these island nations are critical as, irrespective of unknowns and uncertainties, SIDS peoples can expect large-scale changes to their settlements, cultures, knowledges, and identities under climate change, with ensuing impacts on mental health [48]. Although this study also adds to existing studies on emerging emotions in Australia [17,24], by providing a broader view of Oceania, we also capture some of the emotions present in the Pacific Islands context. Existing studies from Oceania have documented feelings of loss, grief, sadness, anger, and stress as a result of acute weather-related disasters such as cyclones [49,50,51,52], and this can be particularly strong for those with lesser capacities to meet basic household needs [53]. Feelings of loss, grief, powerlessness, and uncertainty as a result of “creeping changes” such as sea level rise or drought (which interlinked with fear and worry for family members, the extended society, culture, and the country) [24,34,54] have also emerged. Irrespective of actual climate change impact, discussing anticipated threats and potential losses itself can also have severe mental health and wellbeing impacts in Oceania, including in Tuvalu where daily functioning is becoming impaired [53]. There has also been little investigation of the emotional effects of climate-induced migration in SIDS, which is likely to be a highly stressful experience, especially when forced, and especially among Pacific Islanders who have deep connections to land as a foundation of culture and identity [48]. In Australia, Stanley et al. [10] also found that frustration and anger, compared to eco-anxiety and eco-depression, predicted better mental health outcomes as a greater engagement in pro-climate activism and personal behaviours.

## 2. Methods

This paper draws upon several data sets. The first data set relates to Mt Barney Lodge; an ecotourism business that sits directly outside the Mt Barney National Park in the World Heritage Gondwana area in Queensland, Australia. Mt Barney Lodge was engaged in this study after devastating fires in late 2019/2020 to understand visitor responses to immersing in nature and engaging in a deeply “eco” experience. The three authors took an initial field visit in March 2021, which allowed them to sense and experience the Mt Barney Lodge firsthand. During this visit, they also collaborated with the owners of the Lodge to refine the questionnaire and discuss the recruitment of participants. The questionnaire contained a mix of qualitative and quantitative questions that assessed the impact of nature on wellbeing more broadly, although the aspects drawn on for this study (e.g., questions focused on mental wellbeing) were almost entirely qualitative to gain the rich, in-depth descriptive data of people’s emotions and experiences. In total, the questionnaire had 22 open-ended questions and various close-ended questions, including eight interval scale questions (i.e., Likert scale questions), two yes/no questions, and five nominal questions. The open-ended questions were critical for gaining rich, descriptive data of participants’ emotions, while the Likert scales were useful for measuring the intensity of impacts on wellbeing. The questionnaire was structured by five key sections: (1) overview of participants’ stays at the Lodge, (2) motivations for visiting the Lodge, (3) assessing wellbeing outcomes, including emotional and psychological health before and after staying at the Lodge, (4) understanding participants’ values of nature-based tourism, and (5) final questions gaining background information on participants. Between May and August 2021, questionnaires were administered through the online portal RedCap and participants were recruited via an information sheet and flyer placed in the Lodge reception. Two vouchers of AUD 100 each to stay at Mt Barney Lodge were used as incentives to participate, and although this strategy was not considered coercive, there may have been some participation bias as a result. Seventy-two questionnaires were collected during the recruitment period and all participants were visitors of the Lodge. Quantitative data were analysed using SPSS and qualitative data were analysed through latent content analysis using NVivo. Latent content analysis is a technique used to code social data for its surface and underlying means such that themes in the data can emerge [55]. Ethical approval was granted by Griffith University (approval number 2021/084).

Of the 72 participants who had been a recent visitor to Mt Barney, more were female (71.4%) than male (28.6%). The mean age of participants was 46 years old (youngest was 18 and oldest was 78 years). Slightly under half of all participants had an undergraduate degree (43.1%), followed by a masters (25%), high school completion (16.7%), vocational training (8.3%) and a doctorate (6.9%). The majority of participants indicated that they were “Caucasian” (30.6%), “Australian” (29.2%), “Anglo-Saxon” (6.9%), or had dual heritage. The most prominent areas of work for participants included health (19.4%), followed by education (8.3%), retirement (8.3%) and those that work in the environment field (5.6%), finance (5.6%), or power and water sectors (5.6%).

The second data set is based on an online questionnaire designed to explore and summarise “expert” perspectives on, and emotional responses to, non-economic loss and damage (NELD) in the Pacific Islands. We first identified 360 potential stakeholders, or “experts”, to participate in this study. These are professional “experts” working in the climate change (adaptation, mitigation, loss and damage), development and disaster risk reduction sectors in the region. These “experts” were identified through online searches and from our own networks in the region, and represented different stakeholder groups including government, development partners, civil society, intergovernmental agencies, and relevant others (i.e., research organisations). Carried out between 18 September and 30 October 2020 (6 weeks), the questionnaire contained 27 questions with the majority being open-ended (yielding substantial qualitative data), while the remaining few were close ended (yielding limited quantitative data). The questions centred on knowledge gaps, especially around understandings of what NELD is in the Pacific Islands, types of NELD experiences, and strategies to respond to NELD. The design of the questionnaire was supported by a review of existing literature on NELD categories, which resulted in the survey questions being structured around the following loss categories: human life, human health (physical, mental, emotional), human mobility, territory, cultural heritage, Indigenous knowledge, biodiversity, ecosystem services, sense of place, and social cohesion. Example questions that ascertained understanding around emotional experiences included questions that asked participants to rate how concerned they were about each loss category, and then a follow up question asking for descriptive detail around why they were concerned (i.e., “for categories of actual or potential non-economic loss and damage where you have moderate, high or extensive concern, can you explain this in more detail, giving an example if possible?”). To ensure the research was appropriate and responded to the needs of the region, the questionnaire was piloted in August 2020 with two experts from regional organisations who work directly in loss and damage policy and programming. The questionnaire was then created using Checkbox survey and was emailed to the 360 “experts”, resulting in a response rate of 13% (*n* = 42). Our sampling frame was specific, thus producing a low n-value—only “experts” working in this area, which is limited—and, as such, we believe we gained a rich source of responses with robust representation. As with the first data set, quantitative data were analysed using SPSS and qualitative data were analysed for themes using NVivo [55]. Ethics approval was from the University of Queensland (approval number 2020000640).

Among the 42 participants, there were more males (57.5%) than females (42.5%) and most were in the middle-aged group with the mean age being 42.6 (youngest was 19 and oldest was 63 years). Fiji was the most prominent country of origin and residence for participants, followed by the Cook Islands. Participants also represented Papua New Guinea, Samoa, Vanuatu, Federated States of Micronesia, Solomon Islands, American Samoa, and New Caledonia, but several other Pacific Island countries were not represented by study participants, which is a limitation. A range of institutions were represented by participants, including national governments (35.71%), non-governmental organisations (19.05%), intergovernmental and regional agencies (16.67%), and donors and UN agencies (9.52%). The scale of work for participants ranged from national (47.62%) to regional (33.33%) to local/community (7.14%). Their work also centred around policy development (28.57%), capacity building (11.90%), research (11.90%), on-the-ground practice (9.52%) and education (9.52%).

The third data set that we draw from is interviews with locals living in the Cook Islands. The Cook Islands was chosen given the range of severe weather and climate extreme events that affect the country including tropical cyclones and droughts [56]. Ten structured interviews with 11 participants were conducted between October and November 2020. These interviews were facilitated by staff from the Cook Islands National Council for Women, based in the capital of Avarua on the island of Rarotonga. Given that we wanted to ensure that the voices and perspectives of Cook Islanders in the outer islands—Pa Enua—were included, it was not always possible to undertake in-person interviews (due to phone connections and cost), and thus, the structured interview schedule was sent to the participants to complete. While this presented a limitation for several reasons, including an inability to ask follow-up questions, which was a limitation of all the data sets, the study retained its qualitative approach with rich qualitative data transpiring. The guide was structured into three core sections: (1) participant demographics (e.g., age, gender, religion), (2) livelihoods and values, (3) stories of disaster events and/or climatic changes, (4) experiences and perspectives of intangible loss and damage. The fourth section ascertained in-depth and rich data on emotional experiences of loss, especially with questions such as “When you experienced these losses and/or damages, which ones were of most concern to you or made you the most worried or upset, and why?” or “What future losses and/or damages are you the most worried about?”. The Cook Islands National Council for Women provided the critical role in selecting participants based on their networks. All interview transcripts/written responses were analysed using NVivo, allowing the social data to be coded into prominent “themes” [55]. All participants gave informed consent to participate and the University of Queensland provided the ethical approval for the study (approval number 2020000640).

These 11 interviewees included four women and seven men who all live in the Southern group of islands, except for one from the Northern rural island of Penrhyn/Tongareva. The average age of participants was 55 (youngest was 43 and oldest was 65 years). Participation in community activities differed amongst participants, and most participants identified with diverse Christian denominations. While most participants indicated that they rely on subsistence livelihoods drawing from their gardens, livestock, poultry, and marine resources through fishing, some also pursued various paid work including small businesses and government roles.

We also draw on various spoken, written, and visual art from the Pacific, as art and creative processes have long been critical for sharing, processing, and grappling with and healing from grief, traumatic experiences, and pressing issues [57,58]. Pacifika art presents a unique perspective of cultural practices and often encompasses storylines, emotional expression, and learnings throughout history, including experiences of environmental and climatic change while countering colonial, reductionist representations of Pacific lives and worlds [4,59]. As such, the themes and emotions conveyed in 44 creative/cultural works from across the region were explored, including visual art (*n* = 10), poetry (*n* = 8), mixed media (*n* = 8), song (*n* = 6), film/videography (*n* = 5), documentary (*n* = 4), and theatre performance (*n* = 3). These works were largely found through strategic search techniques, such as browsing online artwork hubs, artist websites, journal papers, and tactical searches of different media types for each country. Latent content analysis was considered the most suitable technique to analyse the data for key themes given its emphasis on revealing underlying means, rather than direct annotation [55].

Although several data sets were drawn upon to allow for cross-comparisons, the sample size of the study remains small and subjective in nature. The results from this study are, therefore, not necessarily transferable or representative of the Oceania experience, yet it has internal validity as it accurately portrays the multiple social realities of our participants who range from professional “experts” working in this field to laypeople. The depth of inquiry enables insight into some of the core emotions and experiences of living in the Anthropocene, which future research can continue to build on and explore by testing in a wider population.

## 3. Results

### 3.1. Acute and Slow-Onset Weather Events: Fear, Stress, Anxiety, and Exhaustion

Feelings of fear, stress, anxiety, and exhaustion emerged in relation to varying intersecting triggers. These included direct impacts from experiencing extreme weather events, direct losses of key livelihood resources as well as the anxiety and worry induced by uncertainty and anticipation for further losses in the future.

The direct impacts of acute-onset events on fear and stress were clear amongst several Pacific Islander participants, reflecting their sudden and devastating nature:

*There is enormous stress already being felt by the higher frequency of El Niño and La Niña, inundation events, droughts etc* (Participant #32, regional study, 2020)

*People were stressed and scared especially for the cyclone as this is a first for everyone* (Participant #9, Cook Islands, 2020)

*I can remember lying in bed that night asleep and waking up to feeling of the walls moving in and then out and hearing the rain just outside our bedroom door on the floor where the roof had come off our home. I have never forgotten that... The worry that the rest of the roof was going to come off, the wind was so loud, and we were in complete darkness without power, it was scary* (Participant #6, Cook Islands, 2020)

The extremes of weather experienced in the Pacific Islands are clear, as participants also emphasised the emotional burden linked to slow-onset events such as drought, especially in response to reduced capacities to meet household and familial needs: “During the drought it was hard not to bring the right food to your family, nerve wrecking and stressful, you just wonder what is happening, sometimes you turn your anger to your family, which is not fair to them... Having no coconuts to feed the pigs was also harder to bear” (Participant #5, Cook Islands, 2020).

Droughts also induce feelings of exhaustion, symbolising the chronic nature of the disaster with prolonged hardships: “When there is drought, we always get unhappy and tired. There is no grass for the goats and no coconuts for the pigs and no water for our house” (Participant #7, Cook Islands, 2020).

Overlapping or consecutive events are also a reality for Pacific Island communities and can have multiplying and cascading impacts on stress, anxiety, and exhaustion as “people are barely recovering from one when another hits” (Participant #32, regional study, 2020). One stakeholder in our regional study summarised that “there are higher levels of PTSD [post-traumatic stress disorder] from the barrage of climate events people have had to endure and the increasingly higher frequency of them” (Participant #31, regional study, 2020). Coupled with stress, however, is also exhaustion. As one participant from the Cook Islands who was exposed to multiple cyclones shared: “...just starting to get some normality to school life then being hit again and again was just exhausting... the whole island was weary, it just felt like we were all exhausted for most of that year” (Participant #3, Cook Islands, 2020). Other Cook Islanders shared how “the effect [of one disaster] after the other, I think makes the struggle harder” and is “extremely stressful” (Participants #5 and #10, Cook Islands, 2020).

A key concern that is prevalent across the Pacific Islands is that “in most cases, psychological assessments are not considered to [be] important during the aftermath of a disaster which can lead to mental health issues and suicides” (Participant #35, regional study, 2020). This is a concern, as several participants highlighted their persisting worries and anxieties post-disaster, especially around future extreme weather events and their unpredictability: “Cyclone is my most worry... as it may happen when the people are not prepared or no warning has been given or might in the middle of the night” (Participant #5, Cook Islands, 2020).

The Mt Barney participants in Australia similarly expressed sentiments that reflected post-traumatic stress disorder, whereby fears and stresses experienced during an extreme weather event are re-felt by specific triggers later. As one participant who experienced the 2019/2020 bushfires in Australia shared, “I still get really anxious when visibility is down or when I smell woodsmoke and don’t know where it’s coming from” (Participant #9, Mt Barney, 2021). For others, levels of anxiety were not necessarily stable or linear, with one participant explaining that they have “[h]igh levels of anxiety over the future, usually peaking at the time of natural [sic] disasters (e.g., floods or bushfires), but also a low level, persistent concern during times of drought, that typically only breaks when there are storms/rains” (Participant #62, Mt Barney, 2021).

### 3.2. Experiencing Loss and Change: Sadness and Grief

Feelings of sadness and grief were also prominent emotional responses across the three studies, particularly in relation to a sense of loss and change. At a Pacific regional scale, there is high “impact of climate change on the mental health of people who see their environment changing and their way of life being very affected” (Participant #31, regional study, 2020). Another participant shared how “it is emotional and crippling to think through some of the NELD issues”, such as those related to health and wellbeing, ways of being, cultural sites and sacred places, Indigenous knowledge, and biodiversity and ecosystem services (Participant #15, regional study, 2020).

Among Australian participants, there was also a deep sense of sadness and grief in response to the damage caused by bushfires, particularly on places they felt connected to:

*Sadness during the Snowy Mountains fires in NSW. This area is where I spend much of my youth, so it was really sad to see it perish. I felt like I was experiencing the same hurt that the environment (trees, wildlife) was—as my memories were embedded in that location* (Participant #60, Mt Barney, 2021)

*Forests I’d grown up walking in were burning, including ancient old rainforests which won’t get a chance to recover… There’s a lot of grief attached to that, and a strong sense of being cut off from the future* (Participant #9, Mt Barney, 2021)

As one participant explained on a regional scale, climate change also poses an existential risk to “home”: “[these are] tragic and avoidable impacts which are causing incredible devastation in Pacific communities... they threaten the existence and way of life of the place I call home” (Participant #13, regional study, 2020). More specifically, participants in the Cook Islands shared their sadness and grief in response to witnessed destruction from cyclones: “the damage it did to my village, it was heartbreaking” and “the aftermath of what happened that night... it was a sad story” (Participants #4 and #5, Cook Islands, 2020).

For other participants in Australia, senses of sadness and grief were more broadly related to anthropogenic changes on the environment as a whole and our way of engaging with nature: “[t]here is an ongoing sense of sadness when, as a society, we manage the natural world so poorly. That so many take what they need and do not give back” (Participant #32, Mt Barney, 2021). After the 2019/2020 bushfires, some participants “have laid awake at night thinking about all the biodiversity loss, climate change and wept” (Participant #16, Mt Barney, 2021), and felt “sad, worried, powerless and so sad for the animals” (Participant #31, Mt Barney, 2021).

Grief is also not restricted to post-loss scenarios, but can also emerge in anticipation of loss and change, particularly in relation to loss of normality and a stable, certain future [8,24]:

*When I was little, I thought of the world as kind of guaranteed—it would always be there—and having that certainty taken away, knowing that a lot of the things I love about the world won’t be there any more, knowing that the world might not be survivable for a lot of people by the time I’m a grown-up—it’s grief, and anger, and fear of how much grief is still to come* (Participant #9, Mt Barney, 2021)

*I would be so saddened if my children could not bring their children to these places. It would be so sad that they could not experience the brilliance of this earth, its rawness and true beauty* (Participant #60, Mt Barney, 2021)

Similarly, in the Cook Islands, a participant shared their concerns over “our people leaving our Ipukarea (country)” in response to change, as this means “no people, no one to care for the land” (Participant #5, Cook Islands, 2020). This is a critical concern as although people “cannot really predict about the future... we have to continue to sustain our way of life” (Participant #10, Cook Islands, 2020). These complex sentiments around sadness and grief have also been reflected in a song titled “Climate Change” by i-Kiribati artist Taki [60] (n.p.) who shared:

*What will be our future?*

*what will be the future of our children?*

*searching for myself*

*seeking a refuge as the world is getting worse day and night*

*my day is so much pain*

*my day is much struggle*

*I cry to my Lord to help me through*

*my people and children*

*my own country*

*stand firm and stay strong until the end of time*

*climate change is so strong.*

### 3.3. Perceived Lack of Agency or Control over Futures: Anticipatory Anxiety, Anger, Frustration, Helplessness and Sadness

It was clear amongst participants that there was a lacking sense of agency or control over futures which was contributing to prominent emotional responses, especially in terms of anticipatory fear and anxiety for their own futures and that of their children:

*I’m always anxious about what the world will look like when I get older and what it will look like for my children when I have them* (Participant #4, Mt Barney, 2021)

*I am worried about climate change, and the tropical cyclones, these are more regular now and more intense* (Participant #1, Cook Islands, 2020)

*I’ve felt strongly anxious about climate change for about four years. I think it just started with there being too much news about the pace of pollution and climate change, which made me feel like it was simultaneously urgent to do something and impossible to know what to do or to do something big enough to have an effect. It was a very panicked feeling* (Participant #9, Mt Barney, 2021)

As a reflection of the existential fears and uncertainty for future Pacific Islanders, one participant also shared: “They [young people] must wonder, “Will I be able to live in my home when I am older, and if not, how can I prepare to live elsewhere? Will I be welcome?”” (Participant #32, regional study, 2020).

In Australia, it was also clear that the perceived lack of agency and control over futures was leading several participants to direct anger and frustration at those in power that they perceived as having more control:

*[I have] a lot of anxiety because it feels like we can’t do anything to stop it, anger because people that can stop it aren’t doing anything* (Participant #14, Mt Barney, 2021)

*I’m angry that I can’t do enough and that people in higher positions of power aren’t doing enough* (Participant #5, Mt Barney, 2021)

Participants emphasised that they felt strong anger and frustration in relation to “the lack of leadership”, the “government’s inability to commit to a decent climate policy” and because “Australia is so focused on fossil fuels when the country could run almost entirely on solar and other green energy sources” (Participants #5, #7 and #3, Mt Barney, 2021). Participants were “equally sad and angry” that “nothing has been done sooner” and that “very little [has been] done to change current practices despite overwhelming evidence that society cannot continue on the path it is on” (Participants #21 and #48, Mt Barney, 2021). Although for Australian participants, it was clear that this anger and anxiety over future uncertainty was a motivating factor for pro-environmental behaviour (e.g., reducing waste, recycling, composting, volunteering, and protesting, and other low impact choices), for others, there was a stronger sense of helplessness and hopelessness:

*[I feel] a lot of anxiety because it feels like we can’t do anything to stop it [climate change]* (Participant #14, Mt Barney, 2021)

*...we have passed the point of no return for damage to our environment and feeling that anything we do to try and change it is never going to be enough* (Participant #40, Mt Barney, 2021)

*Sadness and hopelessness in my ability to stop this. I can’t control this* (Participant #35, Mt Barney, 2021)

*I am just one person, though. These actions [pro-environmental behaviours] do not take away my sadness when I see what humans are doing to the planet all over the world* (Participant #13, Mt Barney 2021)

To be able to cope with their emotions, some participants had turned to “ignor[ing] climate/environmental news” and “try[ing] not to think about it too much”, because “if I don’t know exactly what’s going on, they I won’t suffer the heartache” (Participants #55, #63 and #60, Mt Barney, 2021).

### 3.4. Sense of Injustice: Worry, Helplessness, Anger, and Empowerment

Among the emotions emerging for Pacific Islanders in particular, it was clear that a sense of injustice and lack of power were shaping sentiments of helplessness, worry but also anger and empowerment. One Cook Islander participant, for example, shared:

*I feel worried and helpless, because we know that the natural [sic] disasters are a direct impact from the big country nations with their factory pollution, mining, cutting down trees in the rainforest, strip mining of trees for commercial farming. All this is a direct impact being seen and felt all over our small Pacific Island countries* (Participant #10, Cook Islands, 2020)

In existing Pacifika art, this sense of injustice from international complacency around issues such as climate change has driven strong emotions of anger and betrayal. A poem titled “Rise: from one island to another” by Marshall Islands activist Jetñil-Kijiner [61] (n.p.) and Aka Niviâna from Greenland, reflect this sense of injustice, betrayal, and anger clearly:

*You think you have decades before your home fall beneath tides*

*we have years*

*we have months before you sacrifice us again*

*before you watch from your tv screens and computer screens*

*to see if we will still be breathing*

*while you do nothing*

These sentiments are further emphasised in a theatre performance titled “Te Molimau” by Tokelauan artist Pelesasa [62] (p. 40):

*You want to know what I see when I look out there? I see a graveyard. I see a sinking vaka [boat] and all our ancestors are the passengers. We didn’t do this to ourselves! While people like you were out there living, we were moving our homes inland, we were building walls, heaving water out of the sinking graves of our dead, collecting what remains, and for what? What did you do to stop it?*

This sense environmental violence and injustice was also clearly tied to other senses of injustice, as explored by Samoan poet Siagatonu [63] (n.p.) in their poem Layers:

*There are those who want to talk about climate change, yet don’t want to talk about how those who are affected the most, can’t prioritise it in the first place because prioritising it would mean being forced to pull the layers back and also talk about the poverty, the racism, the injustice, the privilege, the hush money, the hit lists that climate change is operating from, the rounds it makes on earth starting with the most vulnerable. Everyone is affected by climate change, yet some are affected first, but no one cares until it’s affecting them.*

These sentiments of anger and injustice are also tied with a sense of empowerment and resilience, reflecting a drive to persevere with integrity and dignity and a sense of hope and protest. This is evident in Marshall Island writer Leem’s [64] (n.p.) poem titled “More Than Just a Blue Passport”:

*The media turns a blind eye to that in a state of anger fear and panic*

*people act*

*we do not flee*

*we act*

*we will not flee*

*we will act*

*because with each rising wave is our rising resilience*

*and sense of justice and urgency for home, for us I swear I will fight for this grandma*

*I will fight for my family*

*I would fight for my country survival*

*for bigger countries mock us, after they have violated the earth’s virginity with their carbon*

*filled aphrodisiac, digging and pumping off from our mother’s womb relentlessly*

*constantly mocking us*

Tied to this empowerment, resilience and hope is the growing collective identity and sense of solidarity of islands—from the Marshall Islands to Greenland—against global powers, as expressed by Kathy Jetñil-Kijiner [61] (n.p.) and Aka Niviâna:

*My sister*

*I offer you these rocks*

*as a reminder*

*that our lives matter more than their power*

*that life in all form demands*

*the same respect we all give to money*

*that these issues will affect each and every one of us*

*none of us is immune*

*and that each and every one of us has to decide*

*if we*

*will*

*rise*

## 4. Discussion

These four data sets have enabled an exploration of diverse experiences, perspectives, and emotional responses to the Anthropocene from participants across Oceania. First, it was clear that there are strong senses of fear, stress, anxiety, and exhaustion as a result of acute and slow-onset weather events, and that these impacts are heightening and becoming chronic as a result of repeated and prolonged disasters (i.e., drought) [65]. These feelings can emerge (and manifest as anger) as a result of direct impacts from experiencing extreme weather events, such as resource loss and an inability to meet household requirements [51], but they can also emerge in relation to the uncertainty and anticipation for further losses in the future. Often, post-disaster worries and anxieties persisted for lengthy periods, but there is a lack of attention and support for mental health in disaster recovery which can result in worsening mental health issues. Better recognising of emotional impacts also helps us recognise that disaster recovery operates over longer timescales than we might expect, and that the emotional work of recovery can generate new vulnerabilities (e.g., mental health and suicide due to lack of support) [31].

Grief and sadness were also prominent emotional responses in response to loss and change. For Pacific Islanders, these emotions emerged in relation to physical ecological losses (current and anticipated), but also disruptions to intangible aspects such as identity, culture, and way of life [17,53,54]. In Australia and the Pacific, there was also a deep sense of sadness and grief in response to change or losses to places they felt connected to, reflecting existing discussions around grief and the “nostalgia” of losing loved places [9,32,33]. In the Pacific, discussion around sadness and grief and loss of homes was reflective of “solastalgia” specifically, which captures the deep “homesickness” that is felt as home environments are lost or change in ways that are distressing [9,24,32]. These losses and fears can challenge people’s established sense of place and identity [66]. For many in Australia, however, indirect engagements with ecological crises and adverse environmental conditions were also generating strong emotions of sadness and grief, as seen in other studies [13,27]. These are reflective of “macro-worries” and the moral/ethical undertones of grief and sadness (i.e., “other-suffering” moral emotion), which captures the compassion felt for others (whether that be other people, animals, or the environment) [12,18,22,23].

Feelings of grief were also not restricted to post-loss scenarios in the Anthropocene, which is reflective of existing discussions around grief for a stable, pristine, and certain past [8], as well as “anticipatory grief” around loss of a stable future, and then anticipatory anxiety about the grief that is expected to come [14,36,37]. For both the participants in Australia and the Pacific, it was clear that their grief and sadness over the loss of a stable future was self- but also other-directed, the latter being reflective of “moral emotions” and compassion for the suffering of future generations [22,23]. For Pacific Islanders, it was also made clear how the grief over future ecological losses is tied to grief over future losses to culture, livelihoods, place, and ways of life [24,37]. In these ways, in alignment with Cunsolo and Ellis [17], grief is emerging across our case study sites in response to physical ecological losses, disruptions to identity and anticipated future ecological losses, including medium and long-term social and cultural changes.

Participants were grieving the loss of a stable future, which was also tied with a perceived lack of agency or control over futures. This contributed to feelings of anticipatory anxiety, anger, frustration, helplessness, and sadness. Among Pacific Islanders, there were anxieties emerging in relation to the uncertainties of losing homes and migrating [48], although this is not to obscure the long history of Pacific migration and the strength and resilience of existing and future diasporas [4]. For Australian participants, there was a strong sense of anger directed at those in power who they perceived to have more control but implemented little action, making anger elicited by appraisal of others’ (e.g., Australian leaders) norm violations a prominent emotion [20,67]. Although for some Australian participants, anger and anxiety was a motivating factor for pro-environmental behaviour [10,46,68], others felt a sense of helplessness and hopelessness and lack of motivation to adapt behaviour. Research has shown that for worry to lead to adaptive behaviour, people need to perceive the situation as at least somewhat controllable [12,69]. The importance of collective action and trust is relevant here, as to have trust that different societal actors will do their part is a critical aspect of meaning-focused coping, and it makes it seem more worthwhile to do something oneself, while also helping to create constructive forms of hope [12,68]. Beyond feeling helpless, it was also clear how anxieties and sadness can lead to denial and disavowal [7,8].

For Pacific Islanders, there was also a clear sense of injustice and lack of power which shaped sentiments of helplessness and worry, but also anger and empowerment. This reflection draws on the centuries of exploitation of people and nature, and the persisting oppression, domination over, and marginalisation of, Pacific Island nations in the global political economy, which can result in the sense of reduced agency to construct their own futures [70]. Sentiments of anger went beyond milder forms of anger such as irritation and annoyance to reflect stronger forms such as fury and rage, reflecting a sense of moral outrage [18]. Anger and betrayal, as other-condemning moral emotions, highlight the perceived failure of developed countries to uphold Pacific human rights. Pacifika art has also highlighted how environmental violence and other injustices (e.g., legacies of colonialism, imperialism, racism, and political marginalisation) interact and intersect to influence mental and emotional outcomes in the Anthropocene, becoming a stark reminder that the ecological crises of the Anthropocene are rooted in social injustices and associated haunting legacies [4,70].

The productive potential of emotions emerged in our study among some Australians and Pacific Islanders, who draw on anger and anxiety to motivate pro-environmental behaviour, call for change, and inspire hope. In particular, Pacific Islanders and artists were drawing on beliefs and values about justice and human rights to drive motivation to act and to regain a sense of agency over their futures, especially through their resistance to the Anthropocene and engagement in movements for decolonisation and environmental and climate justice [4]. These findings are also reflective of studies that document how anger, in particular, is a key motivator for pro-climate activism and personal behaviour change [10]. In these ways, by making emotions public narratives, there is great potential to release the required energy and the “we-creating capacities” (i.e., our relational ties and responsibilities to others) for change [8,15,16,17,18]. Emotions in Oceania, however, do not always inspire motivation, hope, and action, and can, as seen in other studies, result in a sense of hopelessness, denial, and disavowal [7,8]. There are, therefore, various expressions of and reactions to loss, grief, anxiety, and other emotions, and more discussion is needed on how to find constructive hope and how to shift from collective denial and disavowal to transformative change and effective politics. What is increasingly clear, however, is that expressing, engaging with, and more openly discussing emotions and diverse manifestations of grief in the Anthropocene are a critical first step [8,12,14,15].

## 5. Conclusions

This study provides important insights into understanding the emerging mental and emotional aspects of environmental change and crisis in the Anthropocene. It demonstrates a broad range of emotions, including fear, anxiety, exhaustion, grief, anger, and helplessness, as well as empowerment, through place-specific understandings of impacts and responses. These emotions are emerging in response to acute and slow-onset weather events, experiences of direct loss and change, grappling with a perceived lack of agency or control over futures, and a sense of injustice. This is the first step towards better acknowledging the emotions and grief that are present and emerging in Oceania, such that they can be worked through, and may be used for transformative change, effective politics, and agency over futures. As poignantly shared by one of the participants from Australia, “I don’t want to switch off and ignore my feelings as that means I have given up” (Participant #22, Mt Barney, 2021). Future studies should attempt to further build on, unpack and refine these findings, particularly through disaggregating between different groups within Oceania to explore emotions of the Anthropocene as relevant to vulnerable groups such as women and children [48].

## Data Availability

In accordance with the consent provided by participants on the use of confidential data, data are not available to be shared.
